# Supplementary material for: High temperature methane emissions from Large Igneous Provinces as contributors to late Permian mass extinctions
Source: Nat Commun. 2022 Nov 12;13:6893. doi: 10.1038/s41467-022-34645-3 (PMC9653473; doi:10.1038/s41467-022-34645-3)
Supplement: Supplementary file 1 — Supplementary Information [file 41467_2022_34645_MOESM1_ESM.pdf]

Supplementary Information for

High temperature methane emissions from Large Igneous Provinces as contributors to late Permian mass extinctions

Chengsheng Chen[1, 2, 3], Shengfei Qin[4], Yunpeng Wang[1, 3, #], Greg Holland[5], Peter Wynn[2], Wanxu Zhong[5], Zheng Zhou[2, *]

[1]State Key Laboratory of Organic Geochemistry, Guangzhou Institute of Geochemistry, Chinese Academy of Sciences, Guangzhou 510640, China. [2]Lancaster Environmental Centre, Lancaster University, LA1 4YQ, UK. [3]CAS Center for Excellence in Deep Earth Science, Guangzhou, Guangzhou 510640, China. [4]Research Institute of Petroleum Exploration & Development, PetroChina, Beijing 100083, China. [5]Department of Earth and Environmental Sciences, the University of Manchester, M13 9PL, UK.

[*]Corresponding author. E-mail addresses: z.zhou4@lancaster.ac.uk
[#]Co-corresponding author. E-mail address: wangyp@gig.ac.cn

This file includes:

Supplementary Text

Supplementary Fig. 1 to Supplementary Fig. 6

Supplementary Table 1 to Supplementary Table 6

Supplementary References

Supplementary Text

1. ***Equilibrium of methane clumped isotope and calibration of analytical instrument***

The clumped-isotope composition expressed as $\Delta^{13}CH_3D$ is defined in Stolper et al. (2014), as

$$\Delta^{13}CH_3D = \ln(K) = \ln\frac{[^{13}CH_3D]}{[^{12}CH_3D]} - \ln\frac{[^{13}CH_4]}{[^{12}CH_4]} \quad [4]$$

where four brackets represent the abundance of each methane isotopologue and the equilibrium constant K is a function of temperature for the isotope exchange reaction[1]. In practice, the TILDAS instrument can measure relative molar abundances of the four isotopologues, so that measured $\Delta^{13}CH_3D^*$ values referenced against the stochastic $\Delta^{13}CH_3D$ values estimated from $^{13}CH_4$ and $^{12}CH_3D$ values[1] exist a constant offset $\Delta^{13}CH_3D_{reference}$, that is

$$\Delta^{13}CH_3D_{sample} = \Delta^{13}CH_3D^* - \Delta^{13}CH_3D_{reference} \quad [5]$$

$$\Delta^{13}CH_3D_{sample} = \ln\left(\frac{^{13}R_{sample}}{^{13}R_{reference}}\right) - \ln\left(\frac{^{13}r_{sample}}{^{13}r_{reference}}\right) - \Delta^{13}CH_3D_{reference} \quad [6]$$

where, $^{13}R = {}^{13}CH_3D\ /\ {}^{12}CH_3D$ and $^{13}r = {}^{13}CH_4\ /\ {}^{12}CH_4$ of sample and working reference methane[2]. Equation [6] shows that the constant offset $\Delta^{13}CH_3D_{reference}$ as a non-zero $\Delta^{13}CH_3D$ value can be determined by working reference gases. This means that the working reference gases used for calibration need to be in isotopic equilibria of $\Delta^{13}CH_3D$ compositions at a range of known temperatures[2]. $\Delta^{13}CH_3D$ values are then converted into apparent temperatures (in Kelvin) via the following equation[3] and reported in per mil (‰).

$$\Delta^{13}CH_3D = 1.47348\times10^{19}/T^7 - 2.08648\times10^{17}/T^6 + 1.19810\times10^{15}/T^5 - 3.54757\times10^{12}/T^4 + 5.54476\times10^{9}/T^3 - 3.49294\times10^{6}/T^2 + 8.89370\times10^{2}/T \quad [7]$$

The equilibrium theory of methane clumped isotopes predicts that it can meet thermodynamic equilibrium at specific pyrolysis experiments once experimental conditions match the equilibrium-required conditions[4-6]. For instance, Shuai et al. (2018) confirmed that methane generated by closed-system non-hydrous pyrolysis of coal could yield clumped isotope composition consistent with an isotopic equilibrium of $\Delta^{13}CH_3D$ under either relatively low pyrolysis temperatures (approximately

lower than 500°C) or relatively high temperatures (approximately higher than 600°C)[5]. In this study, we obtained methane in theoretical equilibrium generated between 400 and 500℃ under similar experimental conditions to Shuai et al. (2018). Geochemical characteristics of the generated gases using coal from Jungar basin, China are also comparable to those reported previously in Shuai et al. (2018)[5]. This indicates that gases generated from non-hydrous pyrolysis of coal at specific pyrolysis temperatures can reach thermodynamic equilibrium and be used as standard reference gases for instrument calibration. The process for reference gas generation is described below, while calibration of the TILDAS instrument used in this study is carried out following previously published work[2].

2. *Pyrolysis experiment and reference gas generation*

For spectral characterization and calibration of the TILDAS instrument, we designed a non-hydrous Macro-Scaled Sealed Vessel (MSSV) pyrolysis experiment for obtaining theoretically equilibrated methane gas. Ignite coal was used in the pyrolysis experiment. In order to obtain fresh and un-weathered coal samples, we selected the Jurassic profile in the coal mining area located in southern Jungar basin, Xingjiang, China for sampling, where coal was freshly excavated. The gross geochemical characteristics of the Jungar coal were analyzed using a IFP Rock-Eval 6 and a Finigan Delta XL Plus GC-IRMS. Initial Easy%Ro of the coal was measured by a 3Y-Leica DMP XP microscope[7,8]. The Jungar coal contains total organic carbon (TOC) of 53.3%wt, hydrogen index (HI) of 135 mg HC/g TOC, and T_{max} of 426°C. Its bulk carbon and hydrogen isotopic compositions are -24.5±0.6‰ (PDB) and -133±24‰ (SMOW), respectively. Its initial Easy%Ro is 0.60%, which belongs to ignite coal. These characteristics show that the Jungar coal is comparable to the Yunnan Tertiary coal (ignite coal) reported in a previous study[5], therefore it is suitable for the following experiments in order to obtain methane gas in equilibrium and to conduct calibration.

Aliquots of ~800 mg of 200-meshed coal samples after 48h-60°C drying were weighed and placed in 10 glass vessels. Each vessel was a glass tube (2mm thick, 2.5cm outer diameter, 15cm long) with a narrow single-open end (0.5cm outer diameter, 5cm long). The vessels were flame-sealed under vacuum condition. Then, 10 sealed glass vessels were divided into 5 groups and placed in Muffle tube furnaces at 400°C, 425°C, 450°C, 475°C, 500°C, respectively, and heated for 120h. After pyrolysis, vessels were removed from the furnaces and passively cooled to room temperature. The vessels were then opened using a tube cracker attached to a vacuum line. The produced gas was expanded into an

evacuated 10 mm internally polished refrigeration grade (IPRG) copper tube and split into two aliquots after separating the copper tubes from the middle by using four stainless steel clamps on the ends. A portion of produced gas was also expanded into an evacuated glass bottle and sealed with a silicone cap afterwards. Gases in copper tubes were used for methane clumped isotope measurements and calibration of the TILDAS instrument after gas purification (Supplementary Fig. 4), while gases in glass bottles were used for gas composition and carbon and hydrogen isotope measurements (see Methods). The amount of methane and total gas volume were determined using a high precision manometer by the ideal gas law.

3. *Composition and stable carbon and hydrogen isotope characteristics of major gases generated by pyrolysis experiments*

Methane (CH_4), ethane (C_2H_6), propane (C_3H_8), and carbon dioxide (CO_2) are major gases generated in the pyrolysis experiments (Supplementary Fig. 5a; Supplementary Table 6). Methane is the most abundant recovered gaseous component increasing from 74.61 to 112 ml/g TOC; carbon dioxide is the second most abundant recovered gaseous component decreasing from 93.99 to 68.01 ml/g TOC; ethane is the third most abundant recovered gaseous component decreasing from 13.54 to 0.23 ml/g TOC; other hydrocarbon gases C_3H_8 and $C_{4\text{-}5}$ are always trace components with decreasing trends. The generated gases remain wet (i.e., rich in C_{2+} hydrocarbons) at all temperatures in the pyrolysis experiment, with $C_1/\sum C_{2\text{-}5}$ values ranging from 2 to 9 (Supplementary Fig. 5b; Supplementary Table 6). Measured $\delta^{13}C$ and δD values of methane, ethane, and propane are given in Supplementary Table 6, Supplementary Fig. 5c and 5d. As temperatures of the pyrolysis experiments increase from 400 to 500°C, the $\delta^{13}C$ and δD values of methane evolve from -36.1‰ to -29.3‰ and -303.5‰ to -264.9‰, respectively. Carbon and hydrogen isotope compositions of other gas components all undergo similar increasing trends. Gases generated at high temperatures maintain the $\delta^{13}C$ and δD values in the order of methane < ethane < propane. In addition, methane gas pyrolyzed from the Jungar coal has carbon and hydrogen isotopic values close to the methane gas pyrolyzed from the Yunnan Tertiary coal reported in Shuai, et al. (2018) (Supplementary Fig. 6a). This suggests that reference methane gas generated in this study has reached isotopic equilibrium.

4. *TILDAS instrument calibration by reference gases generated from the pyrolysis experiment and determination of the working reference gas at Lancaster Environment Centre (LEC-1)*

Gases pyrolyzed at temperatures of 400, 425, 475, and 500°C with a duration of 120 h were

purified for methane and analyzed using the TILDAS instrument. Results are shown in Supplementary Table 6 and Supplementary Fig. 6b. Gases pyrolyzed at 450°C were dominated by air suggesting an air contamination during the gas recovery. These samples were therefore omitted from the calibration in this study. Following Eq. [6] and methods in Ono et al. (2014), the $\Delta^{13}CH_3D_{reference}$ value was calculated to be -1.69±0.1‰ (2SE) through the $\Delta^{13}CH_3D^*$ values (Supplementary Table 6). The working reference gas at Lancaster Environment Centre (LEC-1) has a $\Delta^{13}CH_3D$ value of 2.52±0.1‰ (2SE) with $\delta^{13}C$ and δD values of -36.3‰ and -177.2‰ (1SE), respectively (Supplementary Table 6).

(a)

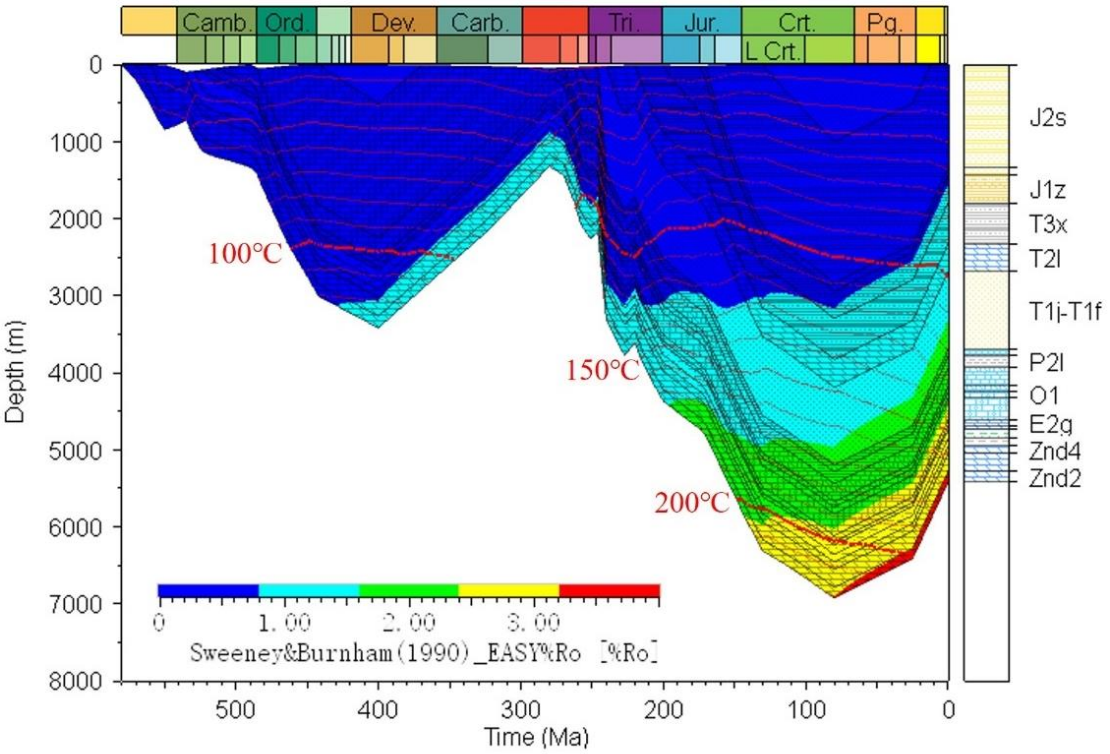

(b)

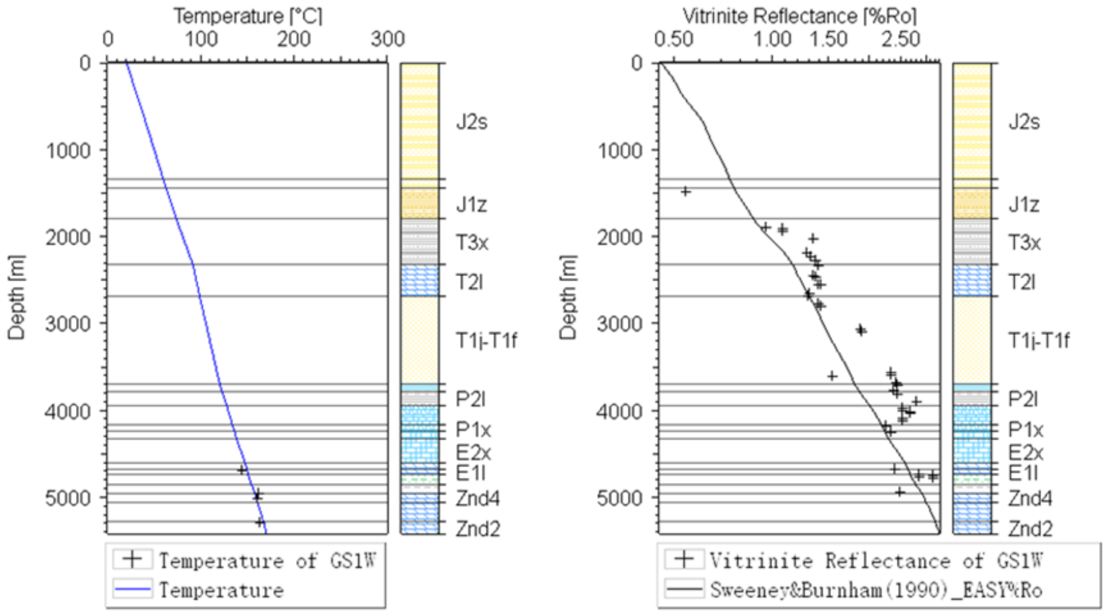

Supplementary Fig. 1. Basin modeling output of model well-GS. (a) Burial-thermal diagram of well-GS. (b) Calibration results of formation temperature and vitrinite reflectance for well-GS.

(a)

(b)

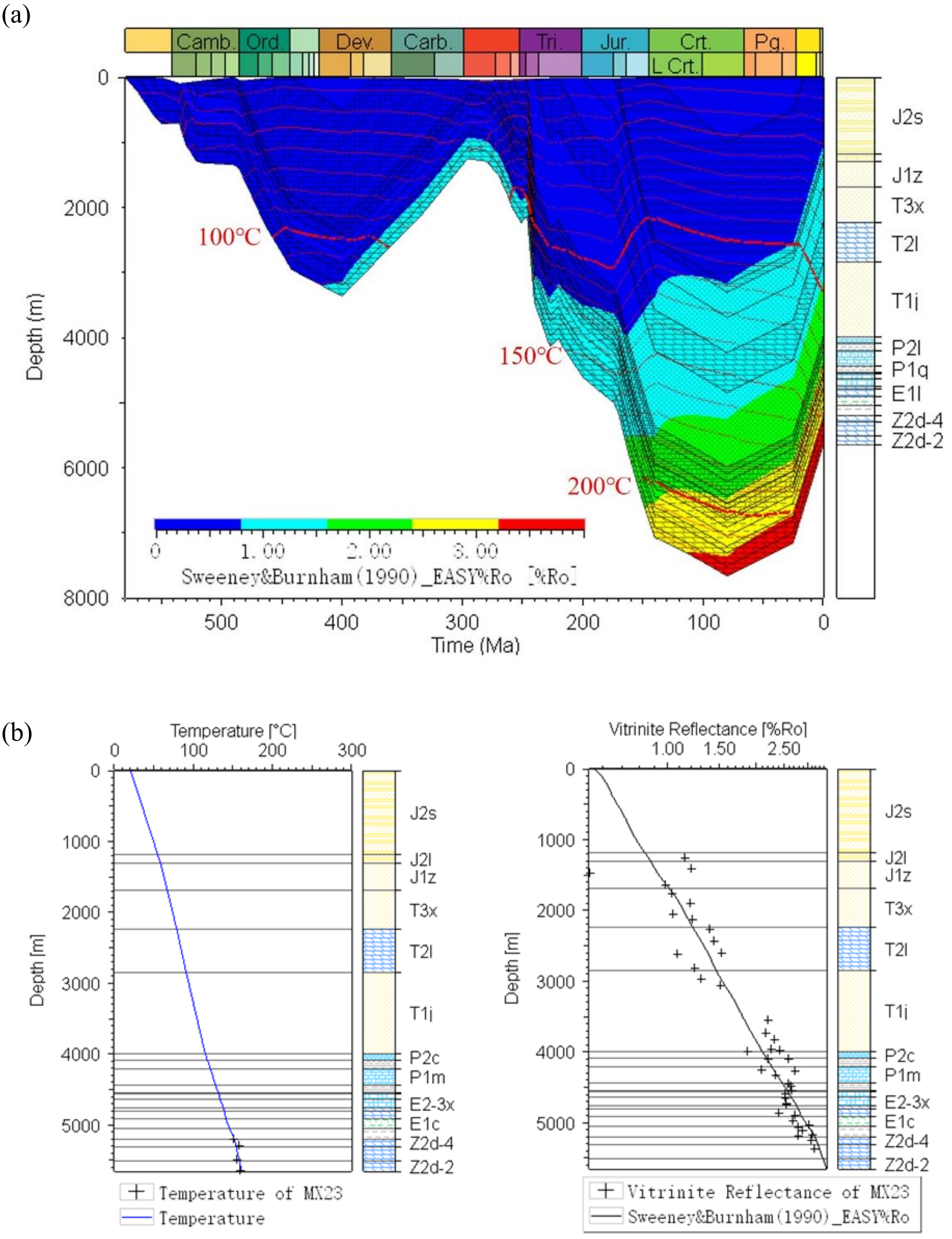

Supplementary Fig. 2. Basin modeling output of model well-MX. (a) Burial-thermal diagram of well-MX**.** (b) Calibration results of formation temperature and vitrinite reflectance for well-MX.

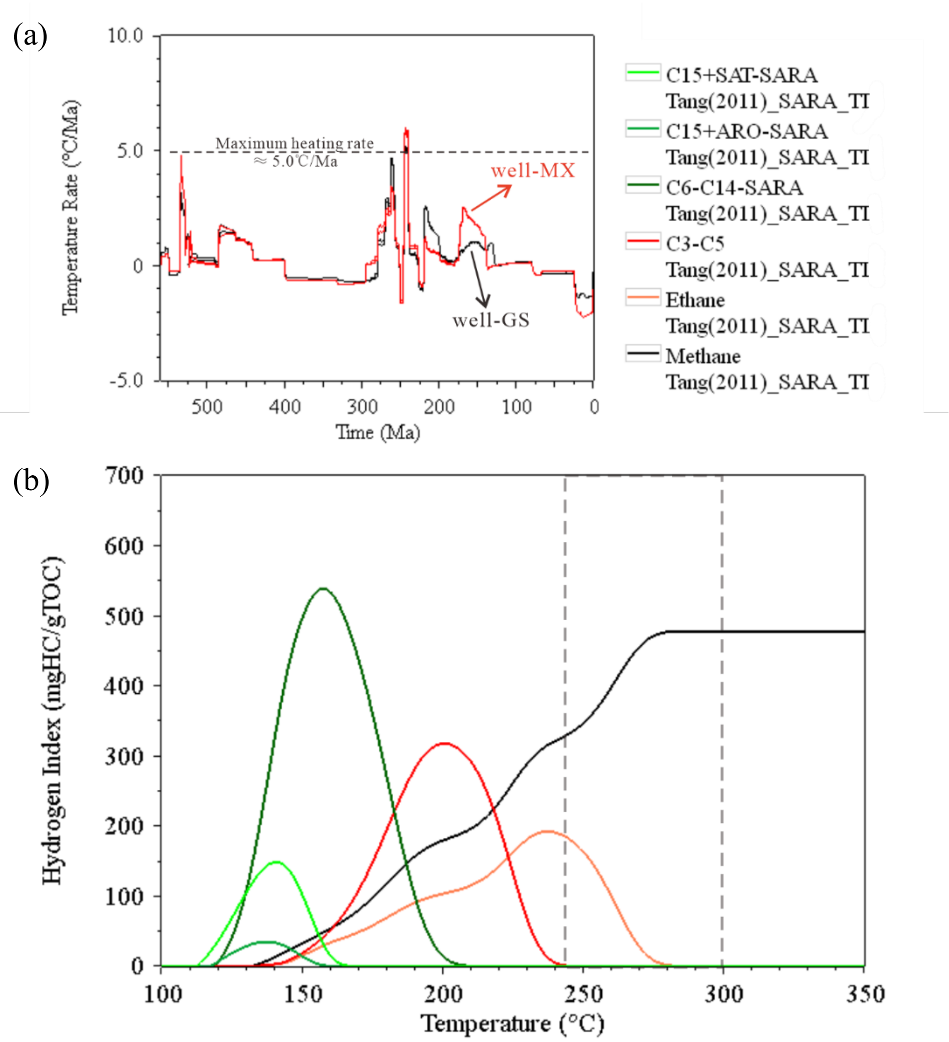

Supplementary Fig. 3. Generation models (a) at a geological heating rate of 5.0°C/Ma (b) based on a kinetic model of Tang(2011)_SARA_TI[9], representing type-I kerogen of the Qiongzhushi (Є$_1q$) source rocks in which the organic matter is mainly of the sapropel type[10-12]. Generally, the peak temperature of oil generation is lower than 160°C; oils (C6+ hydrocarbons) are not produced above 220°C; C3-C5 are not produced above 250°C; Ethane production begins to reduce at 250°C and stops at 280°C.

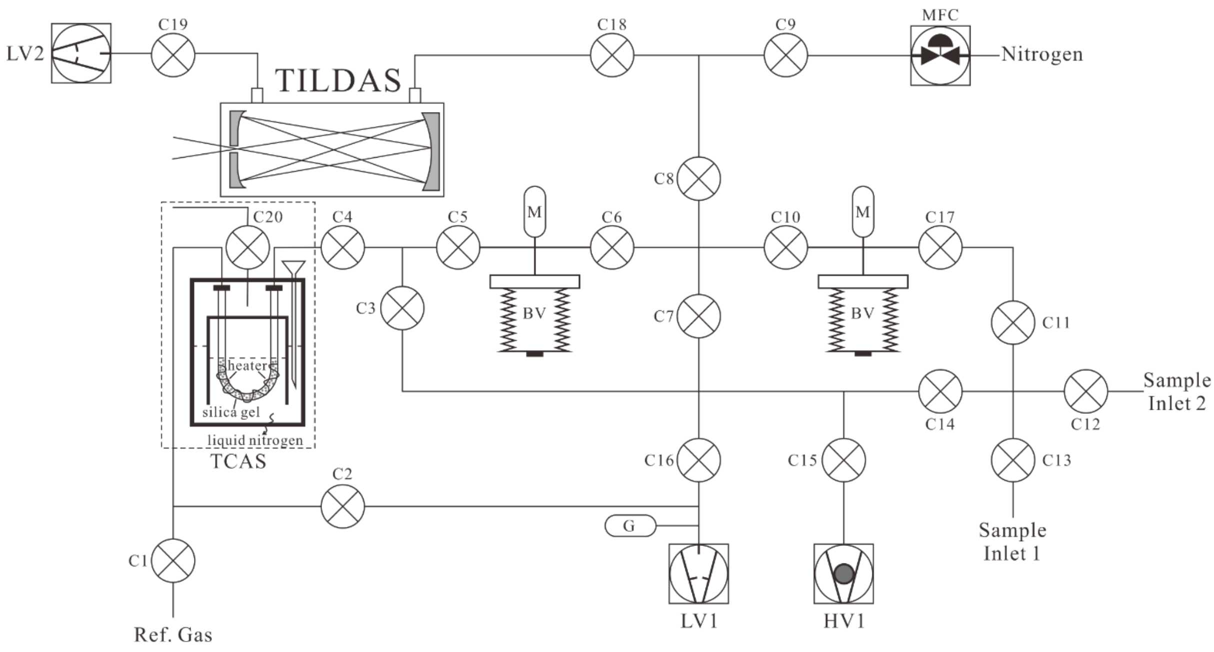

Supplementary Fig. 4. Gas inlet system constructed for the TILDAS instrument at Lancaster Environment Centre. ⊗, automatic valves; M, pressure manometer; G, vacuum gauge; BV, adjustable bellows volume; MFC, mass flow controller; LV, rotary pump for low vacuum; HV, turbo pump for high vacuum; U-trap TCAS, temperature-controlled adsorption system. These components are controlled via custom-built software IBEX Control Centre (Protium MS, UK). Gases can be introduced into the two separate adjustable bellows volumes that can be adjusted between 10-150ml using linear actuators, allowing gas pressure to be controlled within ±0.1 Torr. A portion of sample methane after purification can be introduced into the laser cell by gas expansion from controlling bellow volumes (ca. 10ml). For achieving the capability to completely adsorb sample CH_4, 2.0 grams adsorbent silica gel (0.43m^2/g pore volume; pore size 22Å; 28-200 mesh) was added into the U-trap. The adsorbent temperature can be controlled at a constant point by coordinating the liquid nitrogen interface with the heater. Based on tested adsorbent temperatures for gases, the CH_4 purification process was designed as follows: ①Cool the U-trap to -150℃. ②Expand no less than 30 ml sample gas into the system, at this stage, all hydrocarbon gases are absorbed on the silica gel within 30min (bellow pressures drop below the detection limit to 0.0 Torr), and then pump the system for 10min to evacuate un-adsorbed gases. ③Heat the U-trap to -130℃ and pump the system for 10min to evacuate nitrogen gas (N_2) if it exists. ④heat the U-trap to -100℃, CH_4 is degassed solely within 10min, while heavier gases (e.g. C2+, CO_2 and H_2S) are still frozen on the silica gel; ⑤Tune bellows to introduce purified CH_4 into the TILDAS instrument for analysis; ⑥Heat the U-trap to 250℃ and pump the system for 30min to evacuate residual gases completely under a reserved flow of nitrogen gas for the next test.

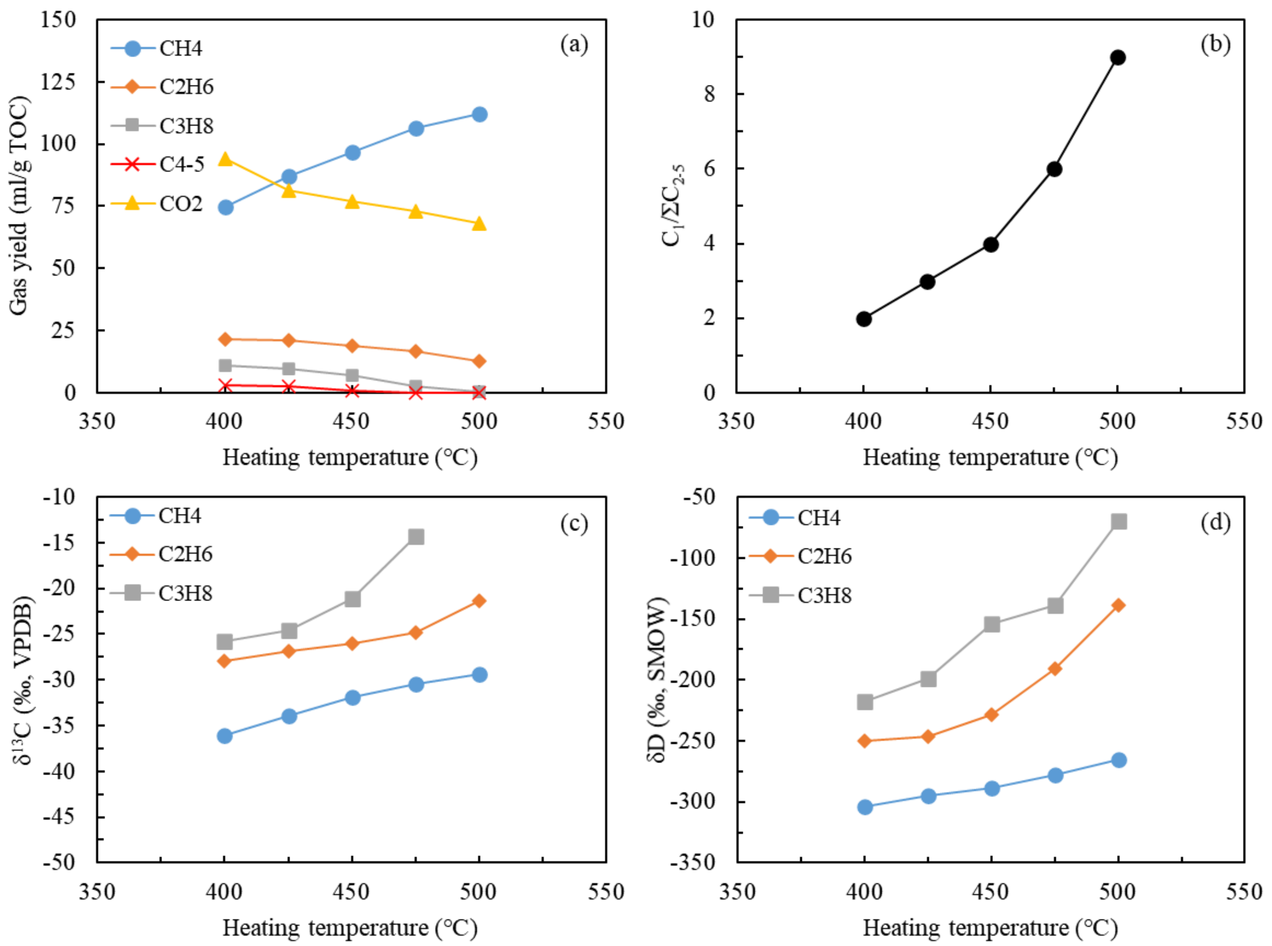

Supplementary Fig. 5. Temperature evolution of (a) yield, (b) dryness, (c) $\delta^{13}C$ value, and (d) δD value of gases generated in the coal pyrolysis experiments.

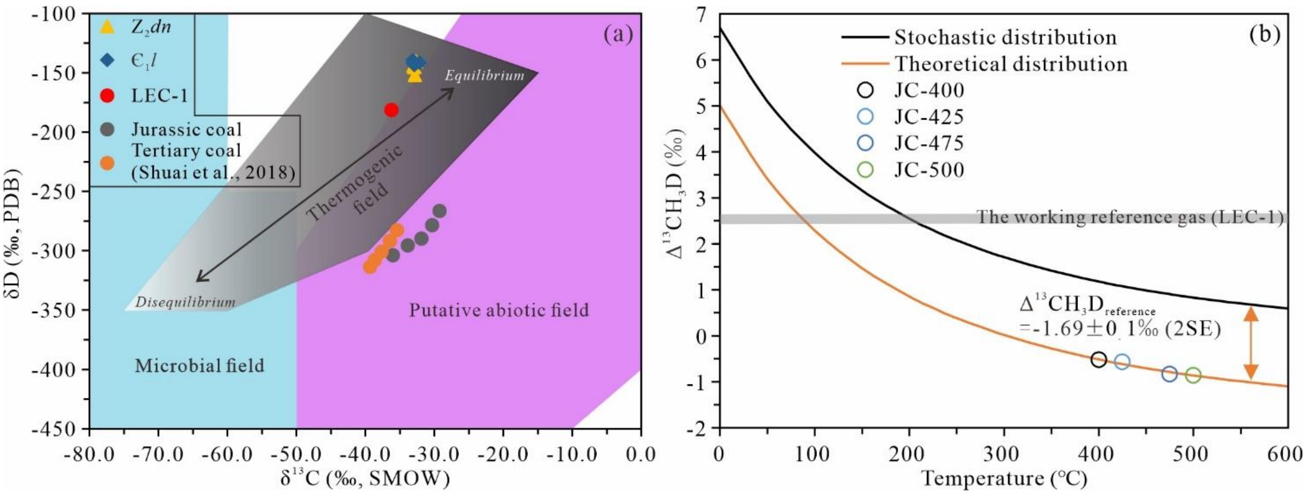

Supplementary Fig. 6. Stable and clumped isotopic characteristics of methane gases. (a) Diagram of $\delta^{13}C$ vs. δD values in samples. Areas filled with different colors show methane gases with forensically diagnostic fields[4]. Black arrows indicate forensic direction of equilibrium and disequilibrium. (b) Experimental calibration of the $\Delta^{13}CH_3D$ thermometer. Open circles represent reference gases pyrolyzed at various temperatures. All error bars are smaller than the symbols. The stochastic distribution (black line) was calculated following Eq. [7]. The theoretical distribution (orange line) was estimated based on the open circles. A constant offset $\Delta^{13}CH_3D_{reference}$ against the black line was determined as -1.69‰, making a cumulative error of ±0.1‰(2SE). The red solid circle represents the working reference gas LEC-1 after calibrating with the offset, which yields $\Delta^{13}CH_3D$ values of 2.52±0.1‰ (2SE), performing the $\delta^{13}C$ and δD values of -36.3±0.1‰ (1SE) and -177.2±0.5‰ (1SE). Methane isotopic compositions plot in the equilibrium thermogenic field (a) with $\delta^{13}C$ and δD values in methane much higher than -60‰ and -300‰, respectively. This indicates that methane is in theoretical equilibrium and the clumped-isotope system is not affected by a biogenic gas component[13,14].

Supplementary Table 1. Major gas species and stable carbon and hydrogen isotopes of gas samples collected from Є$_1$*l* and Z_2*dn* reservoirs in the Anyue gas field, central Sichuan Basin.

Well	Formation	Depth (m)	Gas composition (%)					$C_1/\Sigma C_{2-5}$	$\delta^{13}C$ (‰, PDB)		δD (‰, SMOW)
			CH_4	C_2H_6	CO_2	H_2S	N_2		CH_4	C_2H_6	CH_4
Gaoshi 2	Z_2*dn*	5018-5020	92.14	0.04	6.42	0.70	0.70	2304	-33.1	-27.6	-139
Gaoshi 001-X3	Z_2*dn*	4960-5150	90.11	0.04	8.36	0.97	0.44	2253	-32.7	-28.4	-135
Gaoshi 8	Z_2*dn*	5240-5390	92.00	0.04	6.30	1.51	0.83	2300	-33.0	-28.3	-140
Moxi 22	Z_2*dn*	5400-5500	94.34	0.08	2.65	0.75	2.40	1179	-32.9	-27.5	-147
Gaoshi 1	Z_2*dn*	4956-5130	91.22	0.04	6.35	1.00	1.36	2281	-32.3	-28.1	N.A.
Gaoshi 7	Z_2*dn*	N.A.	N.A.	N.A.	N.A.	N.A.	N.A.	N.A.	N.A.	N.A.	N.A.
Moxi 105	Z_2*dn*	N.A.	N.A.	N.A.	N.A.	N.A.	N.A.	N.A.	N.A.	N.A.	N.A.
Gaoshi 001-H2	Z_2*dn*	N.A.	91.03	0.04	7.02	0.67	0.54	2276	-33.6	N.A.	N.A.
Gaoshi 10	Z_2*dn*	5047-5311	90.58	0.03	6.70	1.11	1.52	3019	-33.4	-28.2	-144
Moxi 108	Z_2*dn*	N.A.	N.A.	N.A.	N.A.	N.A.	N.A.	N.A.	N.A.	N.A.	N.A.
Moxi 022-X1	Z_2*dn*	N.A.	90.28	0.06	2.38	1.40	3.23	1505	N.A.	N.A.	N.A.
Moxi 022-X3	Z_2*dn*	N.A.	90.38	0.08	4.88	1.40	3.23	1130	N.A.	N.A.	N.A.
Moxi 12	Є$_1$*l*	4600-4620	98.16	0.11	0.85	0.00	0.58	892	-33.2	-31.9	-136
Moxi 13	Є$_1$*l*	4570-4590	99.09	0.11	0.15	0.00	0.65	901	-33.2	-31.5	-134
Moxi 009-X1	Є$_1$*l*	4570-4610	95.62	0.12	1.91	0.00	2.35	797	-33.0	-30.8	-138
Moxi 008-H8	Є$_1$*l*	4700-4720	96.85	0.14	1.78	0.00	0.60	692	-32.2	-33.3	-136
Mo 157	Є$_1$*l*	4500-4700	99.03	0.17	0.00	0.00	0.65	583	-33.0	-33.6	-135
Moxi 9	Є$_1$*l*	4987	98.97	0.09	0.05	0.00	0.73	1100	-33.1	-31.6	-136
Gaoshi 2	Є$_1$*l*	N.A.	98.78	0.08	0.40	0.00	0.67	1235	-34.2	-30.5	N.A.
Moxi 204	Є$_1$*l*	4683	99.87	0.13	0.00	0.00	0.00	768	-32.8	-31.5	-138
Moxi 11	Є$_1$*l*	5134	98.73	0.12	0.20	0.00	0.95	823	-33.5	-31.5	-138
Moxi 8	Є$_1$*l*	4864	98.92	0.14	0.21	0.00	0.60	707	-33.6	-32.5	-137
Moxi 008-H1	Є$_1$*l*	N.A.	99.25	0.13	0.22	0.00	0.33	764	-32.6	-32.2	-137
Moxi 008-7-H1	Є$_1$*l*	N.A.	99.11	0.13	0.25	0.00	0.41	762	-34.0	-32.1	-139

Supplementary Table 2. Results for methane clumped isotopes and calculated methane formation temperatures of gas samples collected from $Є_1$*l* and Z_2*dn* reservoirs in the Anyue gas field, central Sichuan Basin.

Well	Formation	$\Delta^{13}CH_3D$ or Δ_{18} (‰)	2SE	Δ_{18}-based T (℃)	2SE
Gaoshi 2	Z_2*dn*	2.09	0.14	248	-16/+17
Gaoshi 001-X3	Z_2*dn*	2.07	0.14	250	-16/+17
Gaoshi 8	Z_2*dn*	2.01	0.15	257	-17/+20
Moxi 22	Z_2*dn*	1.92	0.16	269	-20/+22
Moxi 12	$Є_1$*l*	2.08	0.13	249	-14/+16
Moxi 13	$Є_1$*l*	2.08	0.14	249	-16/+18
Moxi 009-3-X1	$Є_1$*l*	2.10	0.14	246	-15/+17
Moxi 008-H8	$Є_1$*l*	2.07	0.15	250	-17/+19
Mo 157	$Є_1$*l*	2.08	0.14	249	-16/+18

Supplementary Table 3. Noble gas concentrations of gas samples collected from $Є_1l$ and Z_2dn reservoirs in the Anyue gas field, central Sichuan Basin.

Wellhead	Strata	^4He (10^{-5}) cm^3STP/cm^3	Error	^{20}Ne (10^{-10}) cm^3STP/cm^3	Error	^{40}Ar (10^{-5}) cm^3STP/cm^3	Error	^{36}Ar (10^{-9}) cm^3STP/cm^3	Error	40Ar* (10^{-5}) cm^3STP/cm^3	Error
Moxi 13	$Є_1l$	10.30	0.15	7.01	0.12	1.66	0.02	4.67	0.08	1.52	0.03
Moxi 9	$Є_1l$	10.00	0.14	22.50	0.36	1.94	0.03	8.95	0.15	1.67	0.04
Moxi 12	$Є_1l$	9.79	0.14	7.23	0.14	1.91	0.03	5.07	0.10	1.76	0.04
Gaoshi 2	$Є_1l$	14.20	0.20	9.13	0.16	2.70	0.04	4.52	0.10	2.57	0.07
Moxi 204	$Є_1l$	11.00	0.16	11.0	0.19	1.90	0.03	6.09	0.11	1.72	0.04
Moxi 11	$Є_1l$	10.90	0.15	7.71	0.14	1.92	0.03	5.40	0.11	1.76	0.05
Moxi 8	$Є_1l$	10.50	0.15	7.32	0.14	1.72	0.02	5.14	0.10	1.57	0.04
Moxi 008-H1	$Є_1l$	10.10	0.14	8.13	0.14	1.83	0.03	5.43	0.11	1.67	0.04
Moxi 008-7-H1	$Є_1l$	9.67	0.14	7.64	0.14	1.76	0.02	5.26	0.11	1.60	0.04
Wellhead	Strata	^4He (10^{-5}) cm^3STP/cm^3	Error	^{20}Ne (10^{-10}) cm^3STP/cm^3	Error	^{40}Ar (10^{-5}) cm^3STP/cm^3	Error	^{36}Ar (10^{-9}) cm^3STP/cm^3	Error	^{40}Ar* (10^{-5}) cm^3STP/cm^3	Error
Z_2dn-G1											
Gaoshi1	Z_2dn	1.22	0.01	1894	95	17.65	0.22	507	7	2.52	0.05
Gaoshi7	Z_2dn	0.30	0.01	1843	101	13.19	0.23	400	7	1.26	0.03
Moxi105	Z_2dn	1.09	0.01	1814	19	10.91	0.11	323	3	1.27	0.02
Gaoshi001-H2	Z_2dn	1.07	0.01	1772	44	17.00	0.21	538	8	0.94	0.02
Gaoshi001-X3	Z_2dn	1.75	0.02	1486	52	10.56	0.94	353	5	0.03	0.00
Z_2dn-G2											
Gaoshi8	Z_2dn	19.07	0.19	863	82	7.55	0.29	234	10	0.56	0.03
Gaoshi10	Z_2dn	8.44	0.08	924	82	3.35	0.15	102	5	0.30	0.02
Moxi108	Z_2dn	27.52	0.28	0.61	0.06	0.053	0.001	0.181	0.002	0.048	0.001
Moxi22	Z_2dn	30.40	0.30	10.56	0.14	0.091	0.001	1.174	0.013	0.056	0.001
Moxi-022-X1	Z_2dn	28.41	0.28	0.29	0.04	0.066	0.001	0.049	0.001	0.065	0.001
Moxi-022-X3	Z_2dn	25.04	0.25	180	2	1.38	0.01	9.663	0.104	1.09	0.02
Air[15]		0.524		165,000		930		31,420			

Note: ^{40}Ar* denotes non-atmospheric ^{40}Ar. It can be resolved based on the equation $[^{40}Ar^*] = [^{40}Ar]_{tot} \times [1-(^{40}Ar/^{36}Ar)_{air}/(^{40}Ar/^{36}Ar)_{sample}]$, where ^{40}Ar* concentration represents the radiogenic and mantle contribution, and the subscript tot refers to the crustal and total concentrations while the subscripts air and sample refer to the isotopic compositions of the atmosphere and sample, respectively[15]. Data for $Є_1l$ reservoirs are cited from Li et al. (2020)[16].

Supplementary Table 4. Noble gas isotopic ratios of gas samples collected from $Є_1l$ and Z_2dn reservoirs in the Anyue gas field, central Sichuan Basin.

Wellhead	Strata	$^3He/^4He$ (R/Ra)	Error	$^{20}Ne/^{22}Ne$	Error	$^{21}Ne/^{22}Ne$	Error	$^4He/^{20}Ne$	Error	$^{40}Ar/^{36}Ar$	Error	$^{40}Ar^*/^4He$	Error
Moxi 13	$Є_1l$	0.0174	0.0005	9.28	0.21	0.071	0.0022	146933	3302	3555	74	0.148	0.005
Moxi 9	$Є_1l$	0.0115	0.0003	9.74	0.21	0.044	0.0013	44444	945	2168	49	0.167	0.006
Moxi 12	$Є_1l$	0.0130	0.0004	8.96	0.22	0.068	0.0025	135408	3260	3767	95	0.180	0.007
Gaoshi 2	$Є_1l$	0.0256	0.0006	9.23	0.22	0.080	0.0025	155531	3497	5973	159	0.181	0.006
Moxi 204	$Є_1l$	0.0121	0.0003	9.32	0.21	0.056	0.0018	100000	2258	3120	75	0.156	0.005
Moxi 11	$Є_1l$	0.0124	0.0003	9.10	0.22	0.067	0.0022	141375	3221	3556	91	0.161	0.006
Moxi 8	$Є_1l$	0.0127	0.0004	9.87	0.24	0.073	0.0024	143443	3424	3346	76	0.149	0.006
Moxi 008-H1	$Є_1l$	0.0171	0.0004	9.31	0.22	0.068	0.0023	124231	2746	3370	88	0.165	0.006
Moxi 008-7-H1	$Є_1l$	0.0168	0.0004	9.40	0.23	0.070	0.0025	126571	2956	3346	80	0.166	0.006
Wellhead	Strata	$^3He/^4He$ (R/Ra)	Error	$^{20}Ne/^{22}Ne$	Error	$^{21}Ne/^{22}Ne$	Error	$^4He/^{20}Ne$	Error	$^{40}Ar/^{36}Ar$	Error	$^{40}Ar^*/^4He$	Error
Z2dn-G1													
Gaoshi1	Z_2dn	2.00	0.052	9.43	0.48	0.029	0.0005	65	3	348	6	2.055	0.065
Gaoshi7	Z_2dn	1.89	0.050	10.81	0.60	0.028	0.0005	16	1	330	8	4.140	0.151
Moxi105	Z_2dn	1.89	0.057	10.61	1.02	0.030	0.0005	60	1	338	5	1.165	0.039
Gaoshi001-H2	Z_2dn	2.36	0.061	9.08	0.33	0.029	0.0004	61	2	316	6	0.874	0.029
Gaoshi001-X3	Z_2dn	1.37	0.042	10.75	1.03	0.040	0.0011	118	4	300	27	0.020	0.002
Z2dn-G2													
Gaoshi8	Z_2dn	0.382	0.008	12.94	1.16	0.028	0.0005	2210	212	323	18	0.0295	0.0018
Gaoshi10	Z_2dn	0.397	0.029	8.86	0.24	0.028	0.0004	913	82	328	22	0.0358	0.0036
Moxi108	Z_2dn	0.319	0.007	10.33	0.17	0.030	0.0005	4539312	435385	2952	49	0.00174	0.00005
Moxi22	Z_2dn	0.394	0.008	11.45	0.17	0.028	0.0004	287965	4720	778	11	0.00185	0.00005
Moxi-022-X1	Z_2dn	0.418	0.009	7.47	1.06	0.054	0.0016	9962077	1416283	13558	213	0.00229	0.00006
Moxi-022-X3	Z_2dn	0.300	0.008	7.91	0.12	0.030	0.0005	13933	209	1429	21	0.0436	0.0013
Air[15]		1		9.80		0.029		0.288		298.6[17]		1786	

Note: Measured $^{20}Ne/^{22}Ne$ and $^{21}Ne/^{22}Ne$ ratios range from 8.96 to 9.87 and 0.0442 to 0.0799 in $Є_1l$ samples, respectively. Neon isotopic compositions suggest that a two-component mixing process between the air and crust endmembers has occurred in the $Є_1l$ reservoirs, while there is also a minor addition from mantle component or potentially a diffusive fractionation process affecting Ne isotopes[16,18-21]. Measured $^{20}Ne/^{22}Ne$ ratios range from 9.08 to 10.81 in Z_2dn-G2 samples and from 7.47 to 12.94 in Z_2dn-G2 samples, compared with the atmospheric $^{20}Ne/^{22}Ne$ value of 9.80[22]. Measured $^{21}Ne/^{22}Ne$ ratios range from 0.0280 to 0.0400 in Z_2dn-G1 samples and from 0.0276 to 0.0541 in Z_2dn-G2 samples, compared with the atmospheric $^{21}Ne/^{22}Ne$ value of 0.029[22]. Compared to $Є_1l$ samples, the Ne isotopes show unresolved mixing and mass fractionation processes[22,23]. Data for $Є_1l$ reservoir are cited from Li et al. (2020)[16].

Supplementary Table 5. Parameters used in methane emission model and calculating total volume of methane emission from the Anyue gas field.

Formation	data used in equations						calculated results					
	H (m)	C (wt.%)	D (g/cm^3)	R (L/g)	A (km^2)	TGR (m^3)	GGI (m^3/km^2)	TMG (m^3)	TME (m^3)	TME (Gt)	ER (%)	EE (Gt)
Є$_1$*l*	100	1.0	2.8	1.09	22000	4.40×10^{11}	3.05×10^{9}	6.71×10^{13}	6.67×10^{13}	48	99.34%	1345
Z$_2$*dn*	200	1.0	2.8	1.09	22000	5.60×10^{11}	6.10×10^{9}	1.34×10^{14}	1.34×10^{14}	96	99.58%	2696
Є$_1$*l*+Z$_2$*dn*	300	1.0	2.8	1.09	22000	1.00×10^{12}	9.15×10^{9}	2.01×10^{14}	2.00×10^{14}	144	99.50%	4041

H — Reservoir thickness, which values are average[24]

C — Pyrobitumen content, which value is the approximate average of pyrobitumen content about 1.0wt% as its distribution is inhomogeneous[24,25].

D — Rock density, which value is an assumed dolomite density of approximately 2.8 g/cm^3

R — The conversion ratio, which value is the yield ratio of methane and pyrobitumen by artificial thermal simulation of marine origin oils[25]

A — Reservoir area, the limited area of the Anyue gas field[24].

GGI — Gas generation intensity, calculated results from the equation [1] in the Methods.

TMG — Total methane generation, calculated results from the equation [2] in the Methods.

TME — Total methane emission, calculated results from the equation [3] in the Methods.

TGR — Total gas reserves, possible geological reserves in the Anyue gas field exceed one trillion cubic meters[24].

ER — Emission rate, the percentage of TME occupied TMG.

EE — Equivalent CO_2 emission, the global warming potential of CH_4 is 28 times of CO_2 in a 100-year period (IPCC, AR5)[26].

ρ_{CH4} = 0.00072 t/m^3 STP, unit conversion from m^3 to Gt, and 1 Gt = 10^9 t.

Supplementary Table 6. Gas composition and stable carbon and hydrogen isotope characteristics of major gases produced in the pyrolysis experiments (thermally equilibrated methane between 400~500℃ in theory) and working reference gas (LEC-1), and their methane clumped isotope values.

Pyrolysis temperature (℃)	Gas yield (ml/g TOC)					$C_1/\Sigma C_{2-5}$	$\delta^{13}C$ (‰, VPDB)			δD (‰, SMOW)			Methane clumped isotopes (‰)			
	CH_4	C_2H_6	C_3H_8	C_{4-5}	CO_2		CH_4	C_2H_6	C_3H_8	CH_4	C_2H_6	C_3H_8	$\Delta^{13}CH_3D$*	2SE	$\Delta^{13}CH_3D$	2SE
400	74.61	21.61	13.54	2.82	93.99	2	-36.1	-28.0	-25.8	-303.5	-250.2	-218.0	-0.52	0.08	1.17	0.18
425	87.29	20.91	12.23	2.39	81.38	3	-33.9	-26.9	-24.5	-295.2	-246.7	-198.5	-0.57	0.06	1.12	0.16
450	96.63	18.96	7.91	0.89	76.80	4	-31.9	-26.0	-21.1	-289.1	-228.4	-154.2	N.A.	N.A.	N.A.	N.A.
475	106.38	16.59	2.72	0.13	73.08	6	-30.4	-24.9	-14.2	-277.5	-190.6	-138.3	-0.84	0.06	0.85	0.12
500	112.00	12.59	0.23	0.00	68.01	9	-29.3	-21.3	N.A.	-264.9	-138.8	-69.8	-0.86	0.04	0.83	0.11
LEC-1	High-purity methane (>99.99%)					N.A.	-36.3	N.A.	N.A.	-177.2	N.A.	N.A.	0.83	0.04	2.52	0.10

Note: Measured $\Delta^{13}CH_3D$* values before confirming the offset against stochastic distribution; $\Delta^{13}CH_3D$ values after the offset of -1.69±0.1‰ determined.

Supplementary References

1 Stolper, D. A. *et al.* Combined ^{13}C-D and D-D clumping in methane: methods and preliminary results. *Geochim. Cosmochim. Acta* **126**, 169-191 (2014).

2 Ono, S. *et al.* Measurement of a doubly substituted methane isotopologue, $^{13}CH_3D$, by tunable infrared laser direct absorption spectroscopy. *Analytical Chemistry* **86**, 6487-6494 (2014).

3 Eldridge, D. L. *et al.* Comparison of experimental vs theoretical abundances of 13CH3D and 12CH2D2 for isotopically equilibrated systems from 1 to 500 °C. *ACS Earth and Space Chemistry* **3**, 2747-2764 (2019).

4 Dong, G. *et al.* Clumped isotope effects of thermogenic methane formation: Insights from pyrolysis of hydrocarbons. *Geochimica et Cosmochimica Acta* **303**, 159-183 (2021).

5 Shuai, Y. *et al.* Equilibrium and non-equilibrium controls on the abundances of clumped isotopologues of methane during thermogenic formation in laboratory experiments: Implications for the chemistry of pyrolysis and the origins of natural gases. *Geochimica et Cosmochimica Acta* **223**, 159-174 (2018).

6 Stolper, D. A. *et al.* Formation temperatures of thermogenic and biogenic methane. *Science* **344**, 1500-1503 (2014).

7 Sweeney, J. J. & Burnham, A. K. Evaluation of a simple model of vitrinite reflectance based on chemical kinetics. *AAPG Bulletin* **74**, 1559-1570 (1990).

8 Burnham, A. K. Kinetic models of vitrinite, kerogen, and bitumen reflectance. *Organic Geochemistry* **131**, 50-59 (2019).

9 Hantschel, T. & Kauerauf, A. I. *Fundamentals of Basin and Petroleum Systems Modeling*. (Springer, 2009).

10 Zou, C. *et al.* Formation, distribution, resource potential, and discovery of Sinian-Cambrian giant gas field, Sichuan Basin, SW China. *Petroleum Exploration and Development* **41**, 306-325 (2014).

11 Zhu, G., Wang, T., Xie, Z., Xie, B. & Liu, K. Giant gas discovery in the Precambrian deeply buried reservoirs in the Sichuan Basin, China: Implications for gas exploration in oil cratonic basins. *Precambrian Research* **262**, 45-66 (2015).

12 Liu, S. *et al.* The early Cambrian Mianyang-Changning intracratonic sag and its control on petroleum accumulation in the Sichuan Basin, China. *Geofluids* **2017**, 16 (2017).

13 Stolper, D. A. *et al.* The utility of methane clumped isotopes to constrain the origins of methane in natural gas accumulations. *Geological Society, London, Special Publications* **468**, 23-52 (2018).

14 Wang, D. T. *et al.* Nonequilibrium clumped isotope signals in microbial methane. *Science* **348**, 428-431 (2015).

15 Ozima, M. & Podosek, F. A. C. *Noble Gas Geochemistry, 2nd edition*. (University Press, 2002).

16 Li, Y., Qin, S., Wang, Y., Holland, G. & Zhou, Z. Tracing interaction between hydrocarbon and groundwater systems with isotope signatures preserved in the Anyue gas field, central Sichuan Basin, China. *Geochimica et Cosmochimica Acta* **274**, 261-285 (2020).

17 Lee, J.-Y. *et al.* A redetermination of the isotopic abundances of atmospheric Ar. *Geochimica et Cosmochimica Acta* **70**, 4507-4512 (2006).

18 Zhou, Z., Ballentine, C. J., Schoell, M. & Stevens, S. H. Identifying and quantifying natural CO2 sequestration processes over geological timescales: The Jackson Dome CO2 Deposit, USA. *Geochimica et Cosmochimica Acta* **86**, 257-275 (2012).

19 Ballentine, C. J., Burgess, R. & Marty, B. Tracing Fluid Origin, Transport and Interaction in the Crust. *Reviews in Mineralogy and Geochemistry* **47**, 539-614 (2002).

20 Byrne, D. J., Barry, P. H., Lawson, M. & Ballentine, C. J. Determing gas expulsion vs retention during hydrocarbon generation in the Eagle Ford Shale using noble gases. *Geochim. Cosmochim. Acta* **241**, 240-254 (2018).

21 Zhou, Z., Ballentine, C. J., Kipfer, R., Schoell, M. & Thibodeaux, S. Noble gas tracing of groundwater/coalbed methane interaction in the San Juan Basin, USA. *Geochim. Cosmochim. Acta* **69**, 5413-5428 (2005).

22 Ballentine, C. J. & Burnard, P. G. Production, Release and Transport of Noble Gases in the Continental Crust. *Reviews in Mineralogy and Geochemistry* **47**, 481-538 (2002).

23 Byrne, D. J., Barry, P. H., Lawson, M. & Ballentine, C. J. Noble gases in conventional and unconventional petroleum systems. *Geological Society, London, Special Publications* **468**, 127-149 (2018).

24 Wei, G. *et al.* Characteristics and accumulation mode of large-scale Sinian-Cambrian gas reservoirs in the Gaoshiti-Moxi region, Sichuan Basin. *Petroleum Research* **1**, 164-177 (2016).

25 Xiong, Y. *et al.* Formation and evolution of solid bitumen during oil cracking. *Marine and Petroleum Geology* **78**, 70-75 (2016).

26 IPCC. Climate Change 2014: Synthesis Report. Contribution of Working Groups I, II and III to the Fifth Assessment Report of the Intergovernmental Panel on Climate Change. (IPCC, Geneva, Switzerland, 2014).
